# Resonant nanostructures for highly confined and ultra-sensitive surface phonon-polaritons

**DOI:** 10.1038/s41467-020-15767-y

**Published:** 2020-04-20

**Authors:** Alexander M. Dubrovkin, Bo Qiang, Teddy Salim, Donguk Nam, Nikolay I. Zheludev, Qi Jie Wang

**Affiliations:** 1 Centre for Disruptive Photonic Technologies, TPI, SPMS, Nanyang Technological University, Singapore, 637371 Singapore; 2 Centre for OptoElectronics and Biophotonics, School of Electrical and Electronic Engineering, Nanyang Technological University, Singapore, 639798 Singapore; 3 School of Materials Science and Engineering, Nanyang Technological University, Singapore, 639798 Singapore; 4 Optoelectronics Research Centre and Centre for Photonic Metamaterials, University of Southampton, Southampton, SO17 1BJ UK

**Keywords:** Photonic devices, Polaritons, Sub-wavelength optics

## Abstract

Plasmonics on metal-dielectric interfaces was widely seen as the main route for miniaturization of components and interconnect of photonic circuits. However recently, ultra-confined surface phonon-polaritonics in high-index chalcogenide films of nanometric thickness has emerged as an important alternative to plasmonics. Here, using mid-IR near-field imaging we demonstrate tunable surface phonon-polaritons in CMOS-compatible interfaces of few-nm thick germanium on silicon carbide. We show that Ge-SiC resonators with nanoscale footprint can support sheet and edge surface modes excited at the free space wavelength hundred times larger than their physical dimensions. Owing to the surface nature of the modes, the sensitivity of real-space polaritonic patterns provides pathway for local detection of the interface composition change at sub-nanometer level. Such deeply subwavelength resonators are of interest for high-density optoelectronic applications, filters, dispersion control and optical delay devices.

## Abstract (web summary)

Here, the authors demonstrate tunable highly confined surface phonon-polaritons in CMOS-compatible interfaces of nm-thick germanium on silicon carbide. The sensitivity of real-space polaritonic patterns is a pathway for the detection of the interface composition change at sub-nanometer level.

## Introduction

Phonon-polaritons^1,2^ (PhPs) are quasiparticles in the form of photons coupled with electric dipole oscillations of deformable ions in the crystal lattice. They exist in polar crystals at infrared frequencies, hold great promise for low-loss chip-scale nanophotonics^3^ and are considered as an alternative to plasmonics^4^. Being observed in various material systems, PhPs can form electromagnetic waves bound to the bulk^5–13^, surface^14–24^ and edges^25–27^ of the crystal, and can be efficiently controlled by dielectric permittivity and architecture of the device. Moreover, PhPs may carry remarkably large wave vectors compared to the momentum of light travelling in free space. This unique property, also typical to other polaritonic waves^28–33^, has been attracting tremendous attention in the past decade as it enables new platforms for high-density optical integration and strong light–matter interactions at the nanoscale. This opens new opportunities in a number of practical applications, including sub-diffraction elements (resonators^34,35^, waveguides^36^, metamaterials^37^, photonic crystals^38^) and sensing^39,40^.

Highly confined (i.e. large-momentum) PhPs have been extensively studied in natural layered anisotropic two-dimension materials, BN^5–8,11–13,25–27,34–39^ and α-MoO_3_^9,10^, where they form intrinsic modes with hyperbolic dispersion. However, the growth of large-scale high-quality uniform films, capable of supporting intrinsic hyperbolic modes, is a significant challenge^41^, which limits phononic applications of the two-dimension materials to exfoliated-flake-based microscale devices. More recently, another approach of employing nearly isotropic polar crystals with ultra-thin dielectric or semiconductor-coating layers^22,23^ revealed even larger-momentum PhPs at the crystal surface. Such surface PhPs (SPhPs) are practically important as they can exist in numerous combinations of technology-ready polar substrates and coatings, that broadens the scope of their applications. The substrate here serves as the source of SPhPs, while their confinement is achieved by reducing the thickness of the coating layer to extremely sub-diffraction nanometric scale (typically 2–4 orders of magnitude smaller than the wavelength in free space). The remarkable sensitivity of the ultra-confined SPhPs to the thickness and the index of coating layers opens a new avenue for ultra-compact tunable devices and infrared applications at the nanoscale. The cutting-edge performance in practical platform implementations remains a challenge, as reported approaches are limited either by the coating layer quality^22,42^ or the absence of technology to fabricate the high-quality layer at reasonably large scale^23^.

In this work, we demonstrate deeply subwavelength planar resonators supporting extremely confined and tunable SPhPs on group IV semiconductor platform, consisting of silicon carbide substrate coated with a nanometric germanium film of deeply subwavelength thickness. While this system enables ultracompact low-loss on-chip phononic devices with optical features more than two orders smaller than free-space wavelength ($\lambda _0$), it also bears the potential in wafer-scale fabrication compatible with CMOS technology.

## Results

### Few-nm Ge–SiC interfaces for highly confined SPhPs

Using scattering-type scanning near-field optical microscopy (s-SNOM)^43^ we investigate infrared SPhP modes in ultra-thin (thickness $\sim \lambda _0/1500$) germanium slabs and nano-resonators on silicon carbide. As the “gold standard” high-index material for the fabrication of ultra-smooth, wafer-scale and low-loss nanometric coatings, Ge provides excellent conditions for support of high-quality mid-IR SPhP modes. At the same time, it also allows the formation of surface oxidized layer (i.e. Ge oxide)^44,45^, which gives us the opportunity to trace polaritons dynamics under chemical interactions with the environment. s-SNOM technique here provides direct information on the changes in the SPhP wavelength ($\lambda _{\mathrm{p}}$) and mode field distribution, which we model with evolution of the device material composition.

**Fig. 1:**
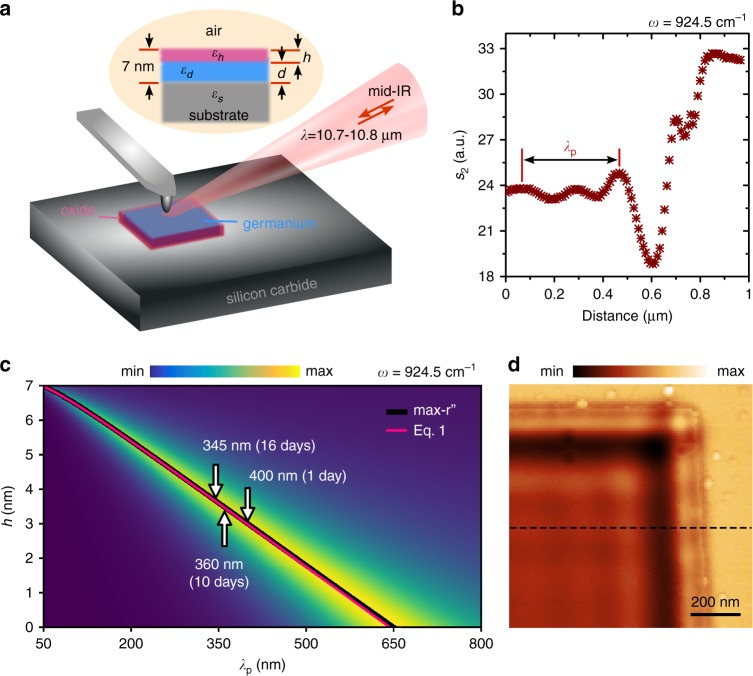
Experimental and theoretical analysis of *λ*_p_ scaling in the case of high confinement. **a** Sketch of the s-SNOM experiment. The inset on top shows schematics of the sample cross-section: a four-layer system consisting of bulk SiC substrate, nanometric germanium and oxide layers, and air. **b** Near-field cross-section taken along black dashed line on image **d**. **c** Plot of the imaginary part of the interface reflection coefficient $r^{\prime\prime}$ for p-polarization calculated at $\omega =$ 924.5 cm^−1^. Black (max-$r^{\prime\prime}$) and pink (based on Eq. (1)) curves depict $\lambda _{\mathrm{p}}$ scaling versus oxide thickness. **d** Near-field image (optical amplitude *s*_2_) recorded at the corner of 7-nm-thick large (10 × 10 μm) germanium microstructure at $\omega =$ 924.5 cm^−1^.

Figure 1a illustrates the schematics of the experiment. Here, the laser radiation within the reststrahlen band of SiC excites SPhPs by means of sub-diffraction optical antenna (metallized tip) for simultaneous launching and detection of polaritonic waves. These waves undergo reflections from the edges of Ge film (or the walls of resonators) forming interference patterns which are mapped together with the topography upon scanning the sample (see “Methods” section for more details). Germanium structures have been fabricated on silicon carbide substrate using electron beam lithography (EBL), followed by 7 nm Ge layer deposition and lift-off processes (see “Methods” section for more details). Owing to its excellent adhesion, the germanium film forms a high-quality interface, which is as smooth as nearly atomically flat substrate (Supplementary Fig. 1). This feature of nanometric germanium films provides the substantial advantage as the polaritons propagation greatly benefits from high-quality interfaces^23^.

Given the ideal Ge–SiC interface, highly confined SPhPs can be described using rigorous^46^ or simplified^23^ dispersion relations for a three-layer system consisting of a negative-permittivity substrate, a positive-permittivity dielectric coating layer of a deep subwavelength thickness, and air. For a 7-nm Ge film on 6H-SiC used in this work, the rigorously calculated SPhP wavelength at $\omega =$ 924.5 cm^−1^ (the excitation frequency mainly used in the experiment) is 643 nm. To describe the actual Ge–SiC interface, however, it is necessary to take into account an ultra-thin germanium oxide layer^44,45^, which is formed on Ge surface upon exposure to the ambient atmosphere. The dispersion of the resulting four-layer system (schematically shown in the inset of Fig. 1a) can be numerically estimated by analyzing of the imaginary part ($r^{\prime\prime}$) of the interface reflection coefficient via transfer matrix method. At a fixed excitation frequency, this numerical calculation produces a value of the SPhP wavelength which depends on the thickness of germanium ($d$) and oxide ($h$) layers. We assume the total thickness of a composite film ($T = h + d$) consisting of germanium and oxide to be fixed at 7 nm (Supplementary Fig. 1) and calculate the SPhP wavelength as a function of the oxide thickness for $\omega =$ 924.5 cm^−1^. The corresponding results are plotted in Fig. 1c. The nearly linear black curve highlights local maxima of $r^{\prime\prime}$ and represents the scaling law of the SPhP wavelength, $\lambda _{\mathrm{p}}(h)$. The sensitivity of polaritons to the oxide thickness is represented by the slope $\Delta \lambda _{\mathrm{p}}/\Delta h$, which ranges as 9.1 ± 0.7 nm per Å along the curve. This number is practically remarkable as it allows locally tracing the composition change of the film at the atomic scale. Indeed, s-SNOM can routinely resolve the spatial optical features of a few 10s nm^47^ (changes in the polariton wavelength in our case), which provides angstrom-level tomographic resolution for the oxide thickness estimation when divided by the slope value. Such resolution is in the range of materials lattice constant—the smallest (bottom-limited) scale of chemical interactions.

We further show (Supplementary Note 1) that simplified analysis of $\lambda _{\mathrm{p}}(h)$ scaling can also be carried out analytically by assuming highly confined nature of SPhP modes ($\lambda _{\mathrm{p}} \ll \lambda _0$). This condition results in the following dispersion relation:

1 $$\alpha _1{\mathrm{{e}}}^{2k_{\mathrm{{p}}}(h - d)} + \alpha _2{\mathrm{{e}}}^{2k_{\mathrm{{p}}}h} + \alpha _3{\mathrm{{e}}}^{ - 2k_{\mathrm{{p}}}d} \simeq \alpha _4$$

where *k*_p_ is the complex polariton wavevector, $\alpha _1\left( \omega \right) = \left( {\varepsilon _{\mathrm{{h}}} - \varepsilon _{\mathrm{{d}}}} \right)\left( {1 - \varepsilon _{\mathrm{{h}}}} \right)\left( {\varepsilon _{\mathrm{{s}}} + \varepsilon _{\mathrm{{d}}}} \right)$, $\alpha _2\left( \omega \right) = \left( {\varepsilon _{\mathrm{{h}}} + \varepsilon _{\mathrm{{d}}}} \right)\left( {1 - \varepsilon _{\mathrm{{h}}}} \right)\left( {\varepsilon _{\mathrm{{d}}} - \varepsilon _{\mathrm{{s}}}} \right)$, $\alpha _3\left( \omega \right) = \left(\! {\varepsilon _{\mathrm{{h}}} + \varepsilon _{\mathrm{{d}}}} \!\right)\left( {1 + \varepsilon _{\mathrm{{h}}}} \right)\left( {\varepsilon _{\mathrm{{s}}} + \varepsilon _{\mathrm{{d}}}} \right)$, $\alpha _4\left( \omega \right) \!=\! \left( {\varepsilon _{\mathrm{{h}}} - \varepsilon _{\mathrm{{d}}}} \right)\left( {1 + \varepsilon _{\mathrm{{h}}}} \right)\left( {\varepsilon _{\mathrm{{s}}} - \varepsilon _{\mathrm{{d}}}} \right)$ and $\varepsilon _{\mathrm{s}}$, $\varepsilon _{\mathrm{{d}}}$, $\varepsilon _{\mathrm{{h}}}$ are permittivities of the substrate (SiC), germanium and oxide layers, respectively. The calculated $\lambda _{\mathrm{{p}}}\left( h \right) = 2\pi /| {{Re} \left( {k_{\mathrm{{p}}}} \right)} |$ solution of Eq. (1) (at $\omega =$ 924.5 cm^−1^ for $T =$ 7 nm) is plotted with pink curve in Fig. 1c, which closely merges with max-$r^{\prime\prime}$ at shorter SPhP wavelengths as expected from the asymptotic. The derived relation would allow direct analysis of the scaling and the dispersion of highly confined SPhPs in nanometric composite films, providing a link between the interface composition change and the polariton wavelength.

To experimentally identify the wavelength of SPhPs, we map polaritons near the edge of an effectively semi-infinite germanium slab (with the size well exceeding SPhP propagation distance) fabricated on a SiC substrate together with nano-resonators. As an example, Fig. 1d shows s-SNOM image recorded at $\omega =$ 924.5 cm^−1^ at the corner of the structure 1 day after the sample fabrication. A typical deep sub-diffraction fringe pattern is observed. The corresponding near-field profile along the black dashed line in Fig. 1d is plotted in Fig. 1b. We note that the global decrease of the near-field amplitude in Ge area compared to bare SiC is a result of a strong change in tip-scattering efficiency between two regions arising from resonant light–matter interactions in s-SNOM tip-SiC system^48^. The extracted SPhP wavelength is 400 nm, which is given by a double value of the fringe period (we use “$\lambda _{\mathrm{p}}/2$” model^5,9,23,49^ for fringes interpretation). By repeating s-SNOM mapping and extraction of $\lambda _{\mathrm{p}}$ at different time after the sample fabrication, we obtain SPhP wavelength dynamics, which is schematically shown with arrows pointing to $\lambda _{\mathrm{p}}$ scaling curve in Fig. 1c. At 1, 10 and 16 days after the fabrication, the polariton wavelength shrinks from 400 to 360 and 345 nm, which corresponds to an increase in SPhP wavelength confinement ($\lambda _0/\lambda _{\mathrm{{p}}}$) from 27 to 30 and 31.4. By matching experimental and calculated values (max-$r^{\prime\prime}$) of $\lambda _{\mathrm{p}}$, we extract the oxide thickness, which increases from 3 to 3.5 and 3.7 nm accordingly. The oxidation of germanium film has been additionally cross-analysed using X-ray photoelectron spectroscopy (XPS) and scanning transmission electron microscopy (STEM), which directly show the chemical presence of germanium oxide at the film surface and its developing with time to the thickness of 3.6 nm (after 16 days) in agreement with the model prediction (Supplementary Note 2).

The discussed atomic-level thickness estimation via SPhPs opens up new possibilities for nanometric film composition tomography, including semiconductor device local screening for the several nanometer technology nodes. At the same time, the discussed interactions provide additional degree of freedom in phonon-polariton nanophotonics, enabling continuous tuning of SPhPs wavelength at a fixed excitation frequency.

### Edge and sheet SPhP modes on Ge–SiC nanoresonators

**Fig. 2:**
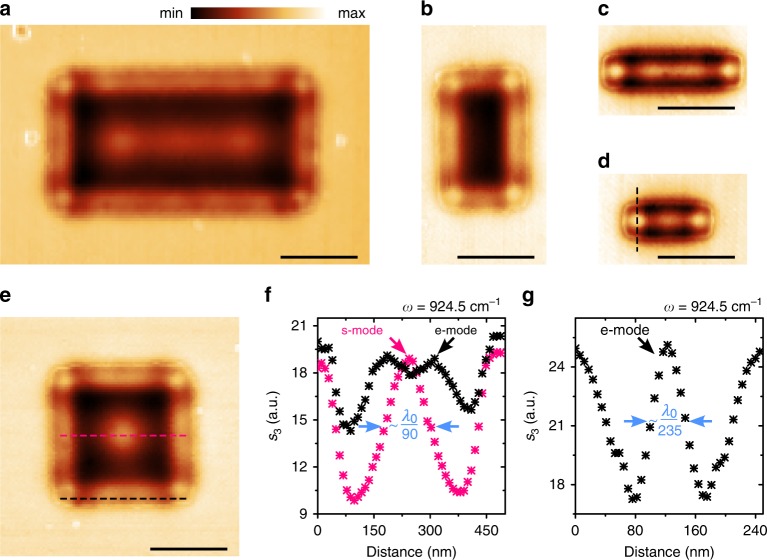
Highly confined surface polariton modes on 7-nm-thick germanium nanoresonators. **a**–**e** s-SNOM images recorded at $\omega =$ 924.5 cm^−1^ for 1000 × 500, 300 × 500, 500 × 100, 300 × 100 and 500 × 500 nm structures correspondingly. **f** Cross-sections along dashed lines in image **e** showing near-field amplitude (*s*_3_) distribution. Pink and black colours represent sheet and edge modes correspondingly. **g** Optical cross-section along black dashed line in image **d**. Colour bar in panel **a** is the same for all images. All scale bars are 300 nm.

As a demonstration of potential phonon-polaritonic devices on a Ge–SiC platform, we perform s-SNOM imaging of Ge rectangular nanoresonators with different sizes. The observed near-field distributions show deep sub-diffraction optical features, represented by number of hot spots both at the inner part of resonators and at its edges (Fig. 2a–e). Such spatial features are usually classified as sheet (s-) and edge (e-) modes correspondingly^50^. Figure 2f, g show optical cross-sections taken along dashed lines across near-field images of a 500 × 500 nm square (Fig. 2e) and a 300 × 100 nm rod (Fig. 2d) resonators. Corresponding estimates for the spatial confinement of the sheet mode at the centre of the square resonator and the edge mode at the corner of the rod resonator are 90 and 235, respectively. These confinement values are defined as the excitation laser wavelength divided by the full width of the observed field distribution peaks at its half maximum. Such a large squeezing of light via SPhPs could enable on-chip optoelectronic devices with ultracompact footprints at the subwavelength scale. Furthermore, we observe tighter spacing of the peaks distribution along the edge compared to the inner part of the resonator sheet (Fig. 2f), which implies the higher-momentum nature of edge modes. We would like to mention that being not necessarily needed to be considered in the concept of tomography, edge modes, however, may provide advantages at extremely small-footprint devices due to its higher confinement. The higher confinement of edge modes has been reported in plasmonic^51^ and hyperbolic^25,27^ material systems, while our results provide the first observation of the phenomenon in nearly isotropic media supporting SPhPs.

### Tuning near-field patterns on Ge–SiC resonator

**Fig. 3:**
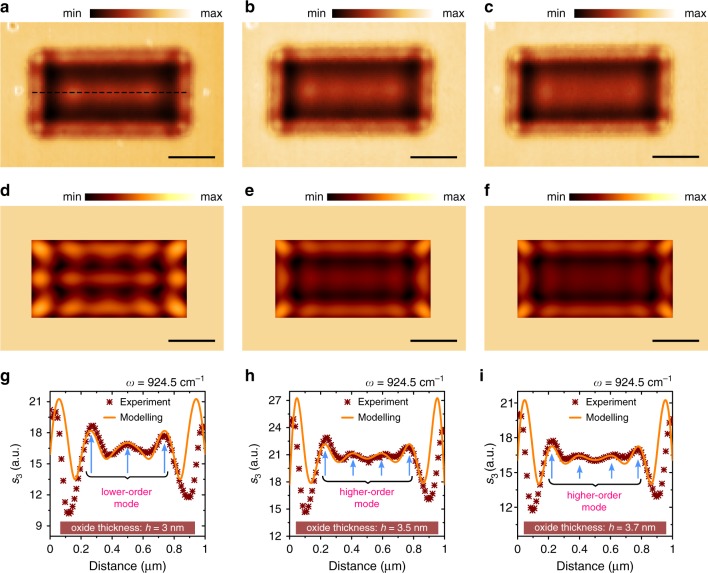
Tuning germanium resonator modes by sub-nm oxidation. **a**–**c** Near-field images of 7-nm thick 1000 × 500 nm resonator recorded at $\omega =$ 924.5 cm^−1^ for different oxide thickness. **d**–**f** Modelling of the modes corresponding to experimental images. **g**–**i** Experimental and modelled near-field distributions along the central horizontal cross-section (as highlighted with dashed line in image **a**) of the resonator modes in data **a**–**f**. Left **a**, **d**, **g**, middle **b**, **e**, **h** and right **c**, **f**, **i** data columns correspond to oxide thickness highlighted with arrows in Fig. 1c, which is labelled on images **g**, **h** and **i**. All scale bars are 300 nm.

Finally, we report the tunability of near-field patterns in the resonator by changing both the composition of the material and the laser excitation frequency. In order to demonstrate the former, we focus on sheet modes as they are better visible in the experiment. To achieve a higher accuracy of tracing the mode dynamics, we perform measurements for the case of the resonator supporting several maximums in the field distribution (1000 × 500 nm rectangular resonator shown previously in Fig. 2a). Figure 3a–c show near-field optical images of the resonator that are subsequently recorded three times: 1, 10 and 16 days after the sample fabrication at $\omega =$ 924.5 cm^−1^. Corresponding experimental data cross-sections are given in Fig. 3g–i. The comparison between Fig. 3g and h, i shows that Ge oxidation as small as ~5 Å in depth leads to complete switching of the sheet mode from a lower to a higher order. This is represented by the appearance of an additional maximum in the field distribution inside the resonator. The experimental results are supported by simulations carried out using the phenomenological model^52,53^, which accounts for all possible reflections of a tip-launched wave in the resonator travelling with the measured polariton wavelength (see “Methods” section for more details). The simulated field distributions are plotted in Fig. 3d–f. Corresponding field profiles in Fig. 3g–i match well with the experimental peaks in the inner part of the resonator but deviate from the measured data at the edges, since the model is constructed for description of sheet modes only, which we aimed to trace in the experiment.

**Fig. 4:**
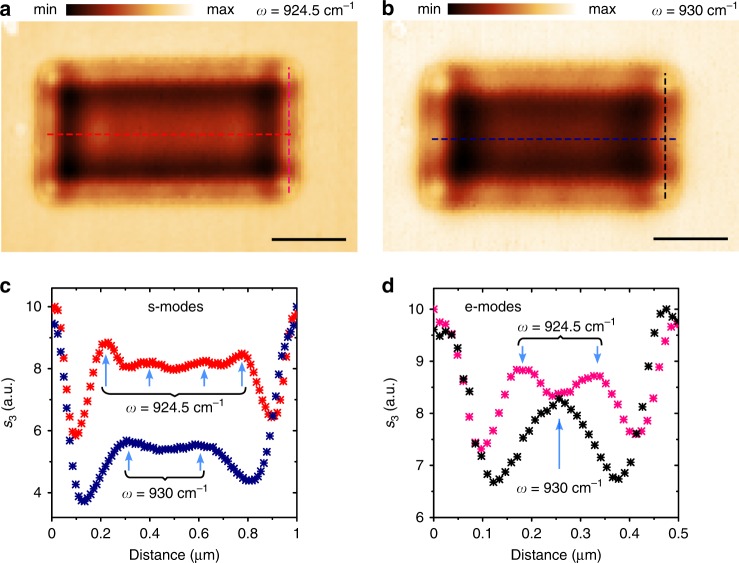
Tuning near-field patterns on germanium resonator by changing the excitation frequency. **a**, **b** s-SNOM images recorded at for 1000 × 500 nm resonator (oxide thickness $h$ = 3.7 nm) at $\omega =$ 924.5 cm^−1^ and $\omega =$ 930 cm^−1^ correspondingly. **c** Normalized near-field cross-sections along horizontal dashed red and blue lines in images **a** and **b** highlighting sheet modes. **d** Normalized near-field cross-sections along vertical dashed pink and black lines in images **a** and **b** highlighting edge modes. Scale bars are 300 nm.

Nanometric Ge–SiC interfaces should hold great potential for efficient spectral tunability of phononic devices as a result of strong dispersion^23^ in the polar crystal. Here, we experimentally demonstrate that tuning of near-field patterns on the resonator is possible by a very small adjustment of the excitation frequency. As seen from Fig. 4a, b, detuning of the laser from $\omega =$ 924.5 cm^−1^ to $\omega =$ 930 cm^−1^ (which is ~0.5% frequency shift) leads to the appearance of a completely new sheet and edge optical field distributions in 1000 × 500 nm structure. The corresponding cross-sections (Fig. 4c), normalized to the field at edges of the resonator for both frequencies, show a transition between four peaks to two peaks in the inner part of the resonator. The edge near-field pattern demonstrates similar transition from a higher to a lower order (Fig. 4d). As an addition study of the spectral tunability, we perform calculations of the polariton wavelength dependence versus oxide thickness at $\omega =$ 930 cm^−1^ (Supplementary Fig. 4), which show an increase of the slope $\Delta \lambda _{\mathrm{{p}}}/\Delta h$ to 14.2 ± 1.2 nm per Å compared to $\omega =$ 924.5 cm^−1^. Using numerical simulations, we also provide an example of refractive index spectroscopic sensing on the introduced Ge–SiC platform (Supplementary Note 3).

## Discussion

We demonstrate that nanometric interfaces between germanium and silicon carbide are capable to support highly confined mid-IR surface phonon-polariton modes at the nanoscale. Owing to the surface nature of the polaritons, the proposed architecture displays good performance at several nanometer semiconductor thicknesses, adding new opportunities for “flatland” optoelectronics. The variety of deep sub-diffraction SPhP modes revealed in Ge–SiC nanoresonators shows the feasibility of potential device footprint miniaturization. Considering high field confinement and the modes tunabilty, the proposed platform could provide a rich playground for integrated nanophotonics and sensing applications.

## Methods

### Samples fabrication

AR-P 6200 resist and AR 600-546 developer (ALLRESIST) were used for EBL process on 6H-SiC wafers. EBL patterns have been written in Helios NanoLab 650 system (FEI). Germanium films were thermally evaporated on the patterned substrates in high vacuum (10^−7^–10^−6^ mTorr) using Univex 250 system (Leybold). The lift-off process was done in AR 600-71 remover.

### Near-field microscopy

A commercial reflection-mode s-SNOM setup (Neaspec) with custom thermally tunable CO_2_ laser (Access Laser) was used for performing infrared nano-imagining. Standard metal-coated tips, Arrow-NCPt (Nanoworld), with ~245–275 kHz oscillation frequency have been utilized as near-field probe in the experiment. Vertically polarized component of the tip-scattered light was selected using a polarizer. Pseudo-heterodyne detection scheme was used for the optical data acquisition. We analysed the tip-scattered signal demodulated at the second (*s*_2_) and the third (*s*_3_) harmonics of the tip oscillation frequency. Low contrast optical cross-sections were averaged by several neighbouring lines to increase signal-to-noise ratio.

### Theoretical calculations

Simulations of the resonator modes are based on ray-optics phenomenological model used for highly confined surface polaritons modelling in literature^52,53^. The tip-launched surface waves, here, propagate circularly outwards until they reach resonator edges, while only normally incident surface waves can be reflected back to the tip position and contribute to the detected signal. The waves continue to oscillate between two pairs of opposite edges, until the energy is fully dissipated due to loss. The resulting complex electric field at each point inside the resonator can be treated as a superposition of four waves and calculated as the summation of four geometric series:

2 $$E = \sum_{j = x_1,x_2,y_1,y_2} {E_j}$$

where $E_j$ represent the total complex electric fields of the mode travelling to and reflected from each edge in *x* and *y* directions from the tip position; $x_1,x_2,y_1,y_2$ are coordinates of the four edges of a rectangular resonator correspondingly. The field of each mode can be expressed as

3 $$E_j = A(1 + r{\mathrm{{e}}}^{2i\beta d_j})\mathop {\sum }\limits_{m = 0}^\infty (r^2{\mathrm{{e}}}^{2i\beta L_j})^m$$

where $A,r,\beta$ denote the amplitude of launched field along each direction, complex reflection coefficient of the mode at the germanium boundary and complex propagation constant of the surface mode. $d_j$ denotes a distance to four edges of the rectangular cavity. $L_j$ is the cavity size in each direction. The real part of $\beta$ is calculated from experimentally determined surface mode wavelength ($\lambda _{\mathrm{p}}$) as $2\pi /\lambda _{\mathrm{p}}$. The magnitudes of $r$ and $A$ are set equal to 1; $d_j$ and $L_j$ are inferred from the tip position inside the cavity and the cavity dimensions. The phase of reflection coefficient and the imaginary part of $\beta$ are used as fitting parameters to reproduce the field distribution obtained in experiment.

The dispersion and scaling relations for the four-layer system have been directly derived from Maxwell’s equations. In calculations, the germanium and germanium oxide are modelled as lossless layers with refractive indexes of 4 and 1.5 correspondingly (Supplementary Note 2); silicon carbide permittivity is used as in literature^23^.

### X-ray photoelectron spectroscopy

The XPS analysis was performed using an AXIS Supra spectrometer (Kratos Analytical) equipped with a hemispherical analyzer and a monochromatic Al K-alpha source (1487 eV) operated at 5 mA and 15 kV. The XPS spectra were acquired from an area of 700 × 300 μm. A 3.1-V bias was applied to the sample to neutralize charge build up on the sample surface. Pass energy 20 eV was used for high-resolution scans acquisition. To obtain the information on the different chemical states of Ge in the sample, curve fitting of the core-level Ge 3*d* peak was performed using the ESCApe™ software (Kratos Analytical). The spectra were charged–corrected based on the fitted Ge 3*d*_5/2_ peak at 29.3 eV. The primary signal from elemental Ge (Ge^0^) was fitted with two components, i.e., Ge 3*d*_5/2_ (29.3 eV) and Ge 3*d*_3/2_ with spin–orbit splitting of 0.58 eV and intensity ratio of 3:2. The Ge suboxide peaks (Ge^1+^, Ge^2+^ and Ge^3+^) were assigned with even spacing of ca. 0.9 eV between the elemental Ge and Ge^4+^ peak. The Ge^4+^ (i.e. GeO_2_) component was left as a free parameter to obtain the best fit.

### Scanning transmission electron microscopy

The Ge–SiC sample cross-section for STEM imaging was prepared in Crossbeam 540 FIB-SEM (ZEISS) system. The sample was protected with ~10 nm Pt layer using sputtering prior to loading in the FIB, and was additionally covered with 200–300 nm of e-beam-induced Pt layer inside the FIB chamber before the cross-section milling. STEM imaging was performed using 2100F (JEOL) microscope operating in dark-field mode.

## Supplementary information

Supplementary Information

## Data Availability

Following a period of embargo, the data from this paper can be obtained from the University of Southampton ePrints research repository, 10.5258/SOTON/D1279.

## Code availability

Following a period of embargo, the code used in this paper can be obtained from the University of Southampton ePrints research repository, 10.5258/SOTON/D1279.
