# Supplementary material for: Resonant nanostructures for highly confined and ultra-sensitive surface phonon-polaritons
Source: Nat Commun. 2020 Apr 20;11:1863. doi: 10.1038/s41467-020-15767-y (PMC7170967; doi:10.1038/s41467-020-15767-y)
Supplement: Supplementary file 1 — Supplementary Information [file 41467_2020_15767_MOESM1_ESM.pdf]

Supplementary Information

Resonant nanostructures for highly-confined and ultra-sensitive surface phonon-polaritons

Alexander M. Dubrovkin[1*], *Bo Qiang*[1,2], *Teddy Salim*[3], *Donguk Nam*[2], *Nikolay I. Zheludev*[1,4*], *Qi Jie Wang*[1,2*]

[1]Centre for Disruptive Photonic Technologies, TPI, SPMS, Nanyang Technological University, 637371 Singapore

[2]Centre for OptoElectronics and Biophotonics, School of Electrical and Electronic Engineering, Nanyang Technological University, 639798 Singapore

[3]School of Materials Science and Engineering, Nanyang Technological University, 639798 Singapore

[4]Optoelectronics Research Centre and Centre for Photonic Metamaterials, University of Southampton, SO17 1BJ, UK

*Correspondence to: dubrovkin@ntu.edu.sg, niz@orc.soton.ac.uk, qjwang@ntu.edu.sg

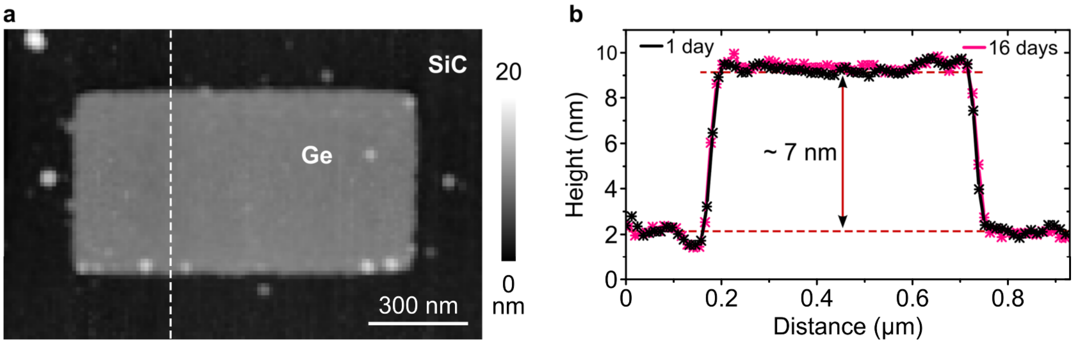

Supplementary Fig. 1 Topography of 1000x500 nm germanium resonator on silicon carbide. **a** Atomic-force microscopic image recorded in s-SNOM together with optical data shown in Fig. 2a in the main text of the article. Sporadic dot-like features are resulted solely from contaminations in EBL step and can be routinely eliminated in technology by optimizing the lithography process. **b** Profiles of the resonator taken along the same cross-section (marked with dashed vertical line in panel (**a**)) at 1 and 16 days respectively.

Supplementary Note 1. Derivation and analysis of the SPhP wavelength scaling relation.

An approximate dispersion relation for a TM-polarized surface-polariton wave in the four-layer system (Eq. (1) in the main text) is derived from Maxwell's equations assuming highly-confined nature of the mode. For solving Maxwell's equations, we expand the approach introduced for two- and three-layer systems[1] to the case of four-layer interface (Supplementary Fig. 2), and additionally applying simplifications to the mode fields before obtaining the final dispersion formula.

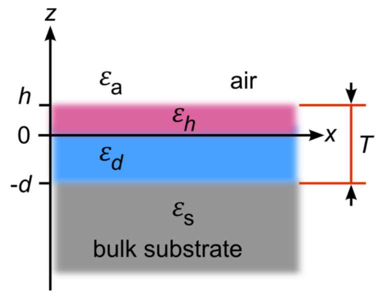

Supplementary Fig. 2 Schematic layout of the four-layer system. A negative-permittivity (ε_s) substrate and air ($\varepsilon_\mathrm{a} = 1$) occupy semi-infinite spaces on the bottom and top of the system. Two intermediate bottom and top layers correspond to germanium and oxide (thickness and permittivity are d, ε_d and h, ε_h correspondingly). T denotes a combined thickness of intermediate layers. y-axis points inwards on the image.

Considering time-harmonic electromagnetic fields oscillating at frequency ω ($\frac{\partial}{\partial t} = -\mathrm{i}\omega$), a TM mode with only H_y magnetic field being nonzero will have the electric component E_x in the form of:

$$E_x = -\mathrm{i}\frac{1}{\omega\varepsilon_0\varepsilon}\frac{\partial H_y}{\partial z}, \tag{1}$$

which directly follows from Maxwell's equations for the media with dielectric permittivity ε; ε_0 denotes dielectric permittivity of vacuum.

Considering Supplementary Eq. (1), the magnetic and electric field components of an interface-bound TM surface polariton wave can be written as a combination of exponentially decaying along z and propagating along x functions (we omit expressions for E_z component as they are not required for the further derivation):

for $z > h$:

$$H_y = A\mathrm{e}^{\mathrm{i}k_\mathrm{p}x}\mathrm{e}^{-q_\mathrm{a}z} \tag{2a}$$

$$E_x = \mathrm{i}A\frac{1}{\omega\varepsilon_0\varepsilon_\mathrm{a}}q_a\mathrm{e}^{\mathrm{i}k_\mathrm{p}x}\mathrm{e}^{-q_\mathrm{a}z} \tag{2b}$$

for $z < -d$:

$$H_y = B\mathrm{e}^{\mathrm{i}k_\mathrm{p}x}\mathrm{e}^{q_\mathrm{s}z} \tag{3a}$$

$$E_x = -\mathrm{i}B\frac{1}{\omega\varepsilon_0\varepsilon_\mathrm{s}}q_\mathrm{s}\mathrm{e}^{\mathrm{i}k_\mathrm{p}x}\mathrm{e}^{q_\mathrm{s}z} \tag{3b}$$

for $-d < z < 0$:

$$H_y = C_d e^{ik_p x} e^{q_d z} + D_d e^{ik_p x} e^{-q_d z} \tag{4a}$$

$$E_x = -iC_d \frac{1}{\omega\varepsilon_0\varepsilon_d} q_d e^{ik_p x} e^{q_d z} + iD_d \frac{1}{\omega\varepsilon_0\varepsilon_d} q_d e^{ik_p x} e^{-q_d z} \tag{4b}$$

for $0 < z < h$:

$$H_y = C_h e^{ik_p x} e^{q_h z} + D_h e^{ik_p x} e^{-q_h z} \tag{5a}$$

$$E_x = -iC_h \frac{1}{\omega\varepsilon_0\varepsilon_h} q_h e^{ik_p x} e^{q_h z} + iD_h \frac{1}{\omega\varepsilon_0\varepsilon_h} q_h e^{ik_p x} e^{-q_h z} \tag{5a}$$

where: $$q_i^2 = k_p^2 - k_0^2\varepsilon_i \tag{6}$$

and $i = \mathrm{a}, h, d, \mathrm{s}$ denote corresponding layers (Supplementary Fig. 2) of the interface; A, B, C_d, D_d C_h, D_h are complex coefficients; k_p is the polariton complex wavevector and k_0 is the wavevector in free space.

Continuity of H_y and E_x at interfaces $z = h$, $z = 0$ and $z = -d$ results in the following system of six linear equations:

for $z = h$:

$$Ae^{-q_a h} = C_h e^{q_h h} + D_h e^{-q_h h} \tag{7a}$$

$$A\frac{q_a}{\varepsilon_a} e^{-q_a h} = -C_h \frac{q_h}{\varepsilon_h} e^{q_h h} + D_h \frac{q_h}{\varepsilon_h} e^{-q_h h} \tag{7b}$$

for $z = 0$:

$$C_h + D_h = C_d + D_d \tag{8a}$$

$$-C_h\frac{q_h}{\varepsilon_h} + D_h\frac{q_h}{\varepsilon_h} = -C_d\frac{q_d}{\varepsilon_d} + D_d\frac{q_d}{\varepsilon_d} \tag{8b}$$

for $z = -d$:

$$Be^{-q_s d} = C_d e^{-q_d d} + D_d e^{q_d d} \tag{9a}$$

$$-B\frac{q_s}{\varepsilon_s} e^{-q_s d} = -C_d \frac{q_d}{\varepsilon_d} e^{-q_d d} + D_d \frac{q_d}{\varepsilon_d} e^{q_d d}. \tag{9b}$$

Assuming large-momentum solutions ($k_p \gg k_0|\sqrt{\varepsilon_i}|$), we reduce Supplementary Eq. (6) to $|q_i| \simeq |k_p|$ and choose $q_i \simeq -k_p$, which corresponds to the propagating negative-index mode[2]. Solving the system (7) – (9) using this assumption, provides a simplified dispersion relation in the form:

$$\alpha_1 e^{2k_p(h-d)} + \alpha_2 e^{2k_p h} + \alpha_3 e^{-2k_p d} \simeq \alpha_4, \tag{10}$$

where $\alpha_1(\omega) = (\varepsilon_h - \varepsilon_d)(\varepsilon_a - \varepsilon_h)(\varepsilon_s + \varepsilon_d)$, $\alpha_2(\omega) = (\varepsilon_h + \varepsilon_d)(\varepsilon_a - \varepsilon_h)(\varepsilon_d - \varepsilon_s)$, $\alpha_3(\omega) = (\varepsilon_h + \varepsilon_d)(\varepsilon_a + \varepsilon_h)(\varepsilon_s + \varepsilon_d)$, $\alpha_4(\omega) = (\varepsilon_h - \varepsilon_d)(\varepsilon_a + \varepsilon_h)(\varepsilon_s - \varepsilon_d)$. Substituting the air-layer permittivity ε_a with 1 in Supplementary Eq. (10) leads to Eq. (1) in the main text of the article.

Supplementary Note 2. Additional analysis of germanium film oxidation.

The oxidation of germanium film has been additionally analysed using X-ray photoelectron spectroscopy (Supplementary Fig. 3a), which directly indicates the chemical presence of both elemental semiconductor and its oxide, as well as the oxide time-dependent development during the sample exposure to the ambient atmosphere. Fitting of the XPS data (see Supplementary Fig. 3b and Methods section in the main text) shows that the oxide is primarily composed by GeO_2 (i.e. Ge^{4+}) component with smaller presence of suboxides (Ge^{3+}, Ge^{2+}, Ge^{1+}). A control measurement of the oxide layer thickness, performed by scanning transmission electron microscopy for the final state of oxidation (i.e. 16 days), gives a value of 3.6 nm (see inset in Supplementary Fig. 3b and Methods section).

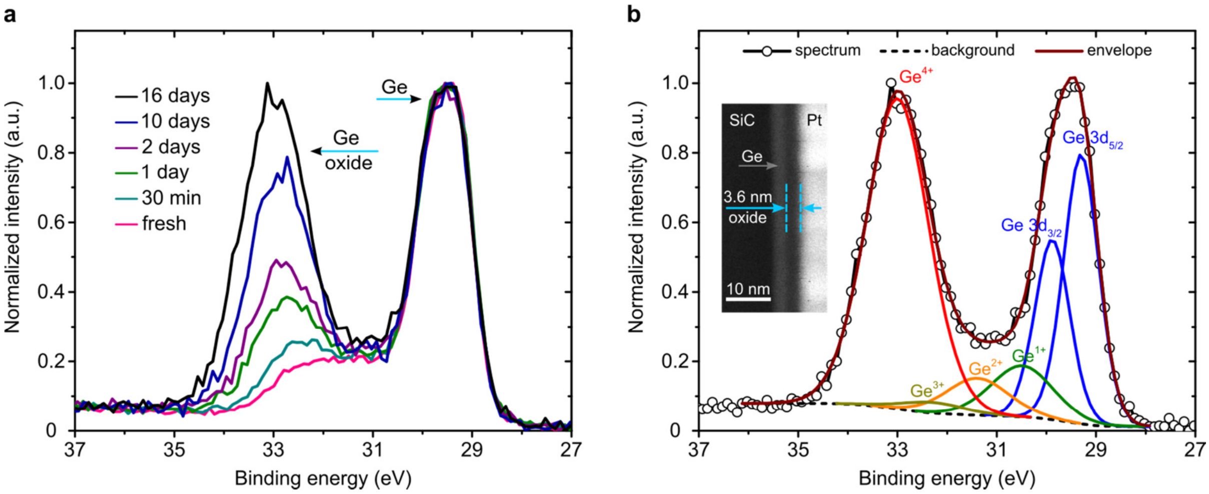

Supplementary Fig. 3 Additional analysis of germanium film oxidation. **a** Normalized XPS spectra recorded after different time of the sample exposure to the ambient atmosphere. The data marked as "fresh" on the panel (**a**) corresponds to less than 5 min exposure time of the sample transferring from the evaporator to the XPS chamber. The data were recorded at 70° emission angle in the XPS system to maximize the photoelectron collection from the sample surface. **b** Fitting of the 16-days data from the panel (**a**). The inset in panel (**b**) shows dark-field STEM image of germanium film vertical cross-section, which consists of (from left to right) SiC substrate, Ge and oxide layers, and protective Pt cap.

The extraction of the oxide thickness from measured phonon polariton wavelength is based on literature data for permittivity of thick crystalline GeO_2 films[3], which we have considered: (1) exactly at the position of the laser line, and (2) by taking an approximate lossless value in the broad transparency window (refractive index of ~1.5), which nearly overlaps with the laser line and covers blue part of the spectrum. While both approaches provide qualitatively comparable results, the approximate lossless permittivity model gives better agreement with control STEM measurement (oxide thickness extracted from 1-day, 10-days and 16-days near-field data are: 2.71, 3.17 and 3.31 nm (based on as-taken from Supplementary ref. 3 permittivity), and 2.99, 3.45 and 3.66 nm (lossless model)). We note that position and strength of germanium oxide infrared absorption peaks may depend on the crystal structure, chemical composition and morphology of the film. We do not exclude possibility that the

infrared properties of nanometric native amorphous oxide layer on germanium may deviate from the reported[3] values.

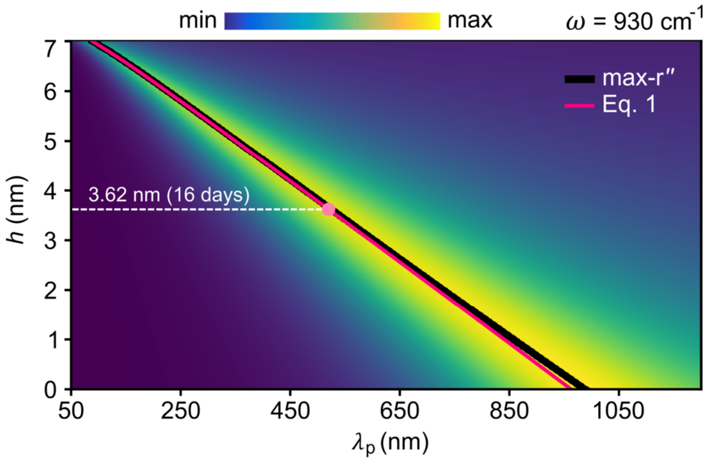

Supplementary Fig. 4 SPhP wavelength scaling calculated for ω = 930 cm^{-1}. The pink dot marks the oxide thickness (3.62 nm) estimated via Eq. (1) based on a control near-field measurement (after 16 days) at this frequency, which is consistent with value obtained from ω = 924.5 cm^{-1} data (i.e. 3.66 nm, as listed in Supplementary Note 2).

Supplementary Note 3. Potential performance of few-nm thick Ge-SiC resonators for refractive index spectroscopic sensing.

Using numerical simulations, we show an example of refractive index sensing on the introduced Ge-SiC platform. The results, summarized in Supplementary Table 1 and Supplementary Fig. 5 below, show that the figure of merit (FOM) for spectroscopic index sensing (FOM = $\Delta\lambda_{\text{res}}/(\Delta n$ ·res_width), the higher the better) on the introduced platform is as good as, if not better than, the state-of-the-art mid-IR devices reported in literature.

Sensing platform	Ge-SiC	Supplementary reference[4]	Supplementary reference[5]	
			split-ring resonator	coupled split-ring resonator
FOM, (RIU^{-1})	2.5	0.65	0.37	1.02

Supplementary Table 1 Comparison of FOM on the Ge-SiC and the platforms published in the literature. The Ge-SiC metamaterial used for the spectroscopic sensing simulations is based on 7-nm thick Ge resonators array. The resonator size used in the simulation is the same as in Fig. 2e of the article main text (500x500 nm) and the array period is 1 µm. For all platforms, the simulations were done in equal conditions using 5 nm thick analyte coating of gradually increasing refractive index, *n*.

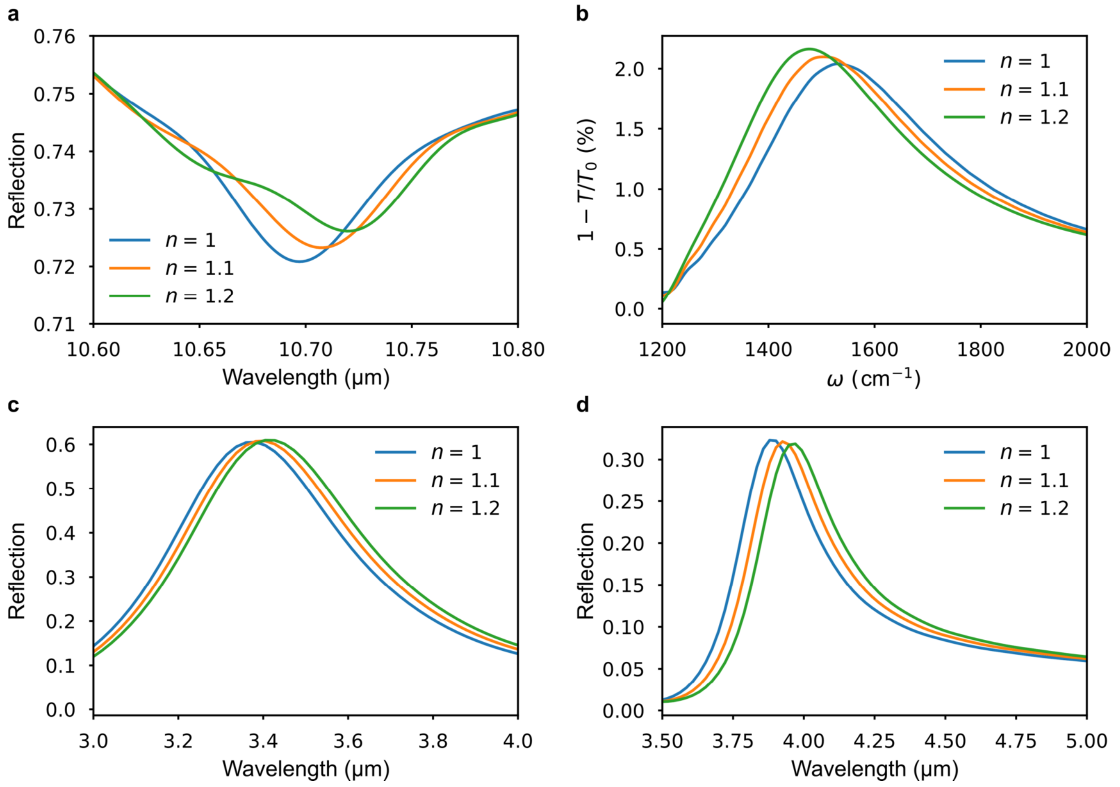

Supplementary Fig. 5 Numerical simulations supporting corresponding data in the Supplementary Table 1. **a** Reflection spectrum shift for the Ge-SiC platform. **b** Extinction spectrum shift for the structure reported in Supplementary reference[4]. **c,d** Reflection spectrum shift for the structure reported in Supplementary reference[5] for cases of split-ring and coupled split-ring based metamaterials respectively. The simulation parameters are the same as listed in the caption of Table 1.

Supplementary References

1. Maier, S. A. *Plasmonics: Fundamentals and Applications* (Springer Science + Business Media, New York, 2007).
2. Dubrovkin, A. M., Qiang, B., Krishnamoorthy, H. N. S., Zheludev, N. I. & Wang, Q. J. Ultra-confined surface phonon polaritons in molecular layers of van der Waals dielectrics. *Nature Commun.* **9**, 1762 (2018).
3. Sun, Y. *et al.* Evaluation of lattice dynamics, infrared optical properties and visible emissions of hexagonal GeO_2 films prepared by liquid phase deposition. *J. Mater. Chem. C* **5**, 12792–12799 (2017).
4. Rodrigo, D. *et al.* Mid-infrared plasmonic biosensing with graphene. *Science* **349**, 165–168 (2015).
5. Pryce, I. M., Kelaita, Y. A., Aydin, K. & Atwater, H. A. Compliant metamaterials for resonantly enhanced infrared absorption spectroscopy and refractive index sensing. *ACS Nano* **5**, 8167–8174 (2011).
